# Limitation of Microbial Processes at Saturation-Level Salinities in a Microbial Mat Covering a Coastal Salt Flat

**DOI:** 10.1128/AEM.00698-21

**Published:** 2021-08-11

**Authors:** Dimitri V. Meier, Andreas J. Greve, Arjun Chennu, Marit R. van Erk, Thirumahal Muthukrishnan, Raeid M. M. Abed, Dagmar Woebken, Dirk de Beer

**Affiliations:** a Department of Microbiology and Ecosystem Science, Centre for Microbiology and Environmental Systems Science, University of Vienna, Vienna, Austria; b Max Planck Institute for Marine Microbiology, Bremen, Germany; c Leibniz Centre for Tropical Marine Research, Bremen, Germany; d Biology Department, College of Science, Sultan Qaboos University, Muscat, Sultanate of Oman; University of Michigan-Ann Arbor

**Keywords:** biofilm biology, element cycles and biogeochemical processes, extremophiles/extremophily, microbial communities, microbiology of unexplored habitats, primary and secondary production, uncultured microbes

## Abstract

Hypersaline microbial mats are dense microbial ecosystems capable of performing complete element cycling and are considered analogs of early Earth and hypothetical extraterrestrial ecosystems. We studied the functionality and limits of key biogeochemical processes, such as photosynthesis, aerobic respiration, and sulfur cycling, in salt crust-covered microbial mats from a tidal flat at the coast of Oman. We measured light, oxygen, and sulfide microprofiles as well as sulfate reduction rates at salt saturation and in flood conditions and determined fine-scale stratification of pigments, biomass, and microbial taxa in the resident microbial community. The salt crust did not protect the mats against irradiation or evaporation. Although some oxygen production was measurable at salinities of ≤30% (wt/vol) *in situ*, at saturation-level salinity (40%), oxygenic photosynthesis was completely inhibited and only resumed 2 days after reducing the porewater salinity to 12%. Aerobic respiration and active sulfur cycling occurred at low rates under salt saturation and increased strongly upon salinity reduction. Apart from high relative abundances of *Chloroflexi*, photoheterotrophic *Alphaproteobacteria*, *Bacteroidetes*, and *Archaea*, the mat contained a distinct layer harboring filamentous *Cyanobacteria*, which is unusual for such high salinities. Our results show that the diverse microbial community inhabiting this salt flat mat ultimately depends on periodic salt dilution to be self-sustaining and is rather adapted to merely survive salt saturation than to thrive under the salt crust.

**IMPORTANCE** Due to their abilities to survive intense radiation and low water availability, hypersaline microbial mats are often suggested to be analogs of potential extraterrestrial life. However, even the limitations imposed on microbial processes by saturation-level salinity found on Earth have rarely been studied *in situ*. While abundance and diversity of microbial life in salt-saturated environments are well documented, most of our knowledge on process limitations stems from culture-based studies, few *in situ* studies, and theoretical calculations. In particular, oxygenic photosynthesis has barely been explored beyond 5 M NaCl (28% wt/vol). By applying a variety of biogeochemical and molecular methods, we show that despite abundance of photoautotrophic microorganisms, oxygenic photosynthesis is inhibited in salt-crust-covered microbial mats at saturation salinities, while rates of other energy generation processes are decreased several-fold. Hence, the complete element cycling required for self-sustaining microbial communities only occurs at lower salt concentrations.

## INTRODUCTION

Microbial mats are densely populated, stratified microbial assemblages performing complete element cycling, perfect for studying microbial interactions and the development of complex microbial ecosystems. Microbial mats are often found in “extreme” habitats with high UV radiation, high temperatures, and high salinity, such as solar salterns (reviewed in references 1 and 2) and coastal sabkhas, intertidal or supralittoral zones with accumulated evaporites as a result of an arid climate (3). Traces of microbial mats are found early in the rock record, and they may also represent the last surviving ecosystems on a future Earth (4) or be potential analogs for surface communities on other planets (5). In these microbial communities, primary production by photosynthesis is balanced by a suite of degradation processes (6), including hydrolysis of organics, aerobic respiration, and sulfate reduction. The latter process can drive an active sulfur cycle, where sulfide produced by sulfate reduction is oxidized by phototrophs and aerobic sulfur oxidizers (7, 8).

In hypersaline environments, microbial cells need to actively counter the osmotic stress imposed by high salinity by either accumulating small organic molecules (osmolytes) or potassium ions (reviewed in reference 9) in order to retain water inside the cell. These stress tolerance strategies are energetically costly and theoretically can impose thermodynamic limits on the type of possible metabolic processes (10) when the cost of growth plus salt adaptation exceed the energy that can be generated by metabolic activity. Processes with low energy yields that were doubted to support growth at high salt concentrations (salinity above 15%) include sulfate reduction, nitrification, and methanogenesis (10). However, with more recent reports of sulfate reduction (11, 12), ammonia oxidation (13), and even methanogenesis (14) at high salinities, it has been argued that hypersaline environments can be considered “thermodynamically moderate,” allowing for active complete element cycling and thus can represent self-sustaining ecosystems (15).

The key to a truly self-sustaining ecosystem with sunlight as the only perennial energy source is primary production of organic matter by oxygenic phototrophs. While prominent layers of filamentous cyanobacteria can be observed in moderately hypersaline habitats, the diversity of oxygenic phototrophs diminishes at higher salinity (16) and eukaryotic *Dunaliella* algae are often referred to as the sole primary producer in salt-saturated brines (17). However, detection of microorganisms by microscopy or molecular markers (DNA, pigments, lipids) does not provide information on their metabolic activity, and it is well known that microorganisms can survive long periods of unfavorable conditions in dormant states (18–20). While active growth of heterotrophic halophilic archaea and bacteria in saturated brines is well known, less data are available on purely photosynthetic organisms and possible limits of their activity. Even in *Dunaliella* algae, photosynthesis rates have been reported only up to 3 M NaCl concentrations (ca. 18% wt/vol salinity) with some studies showing a decrease of photosynthesis rates (21, 22) and others showing an enhancement (23) at increasing salinities. However, the growth of *Dunaliella* decreases drastically when approaching salt saturation (24, 25). Although oxygenic photosynthesis is not thermodynamically limited at high salt concentrations (10), salinity shift experiments with microbial mats indicated a kinetic inhibition of oxygenic photosynthesis by decreased oxygen solubility (26, 27).

Conversely, saturation-level salinity might also provide some life-preserving effect. Already Baas Becking suggested that crusts formed by precipitated salt can protect underlying life against heat and intense light (28). Crystalline halite was further observed to facilitate condensation of water from the air and slow down its evaporation, thus forming a refuge for phototrophic microbial life (13, 29). Although measuring the *in situ* photosynthetic activity in such salt-covered habitats remains a challenge, Davila et al. provided indications that, in halite-colonizing communities of the Atacama Desert, it might be performed by very few unicellular *Cyanobacteria* at low rates (13).

Microbial mats containing filamentous *Cyanobacteria* were found under a layer of crystalline salt at a tidal flat in Oman, with a distinct microbial community composition at the upper tide line, suggesting an adaptation of these microorganisms to high salinity (30). We investigated the limitations of metabolic processes to high salt stress in this salt-saturated environment, with special focus on photosynthetic primary production and tested the hypothesis that salt crust may protect microbial communities against desiccation, heat, and excessive irradiation. We measured light penetration, oxygenic photosynthesis, respiration, and sulfate reduction in salt crust-covered mats at saturation-level salinity and after mimicking a natural flooding event leading to dissolution of the salt crust and reduction of salinity in the mats. The microbial composition of individual mat layers was determined by microscopy, pigment, and 16S rRNA gene amplicon analysis.

## RESULTS

### Geochemistry and mat characteristics.

**FIG 1:**
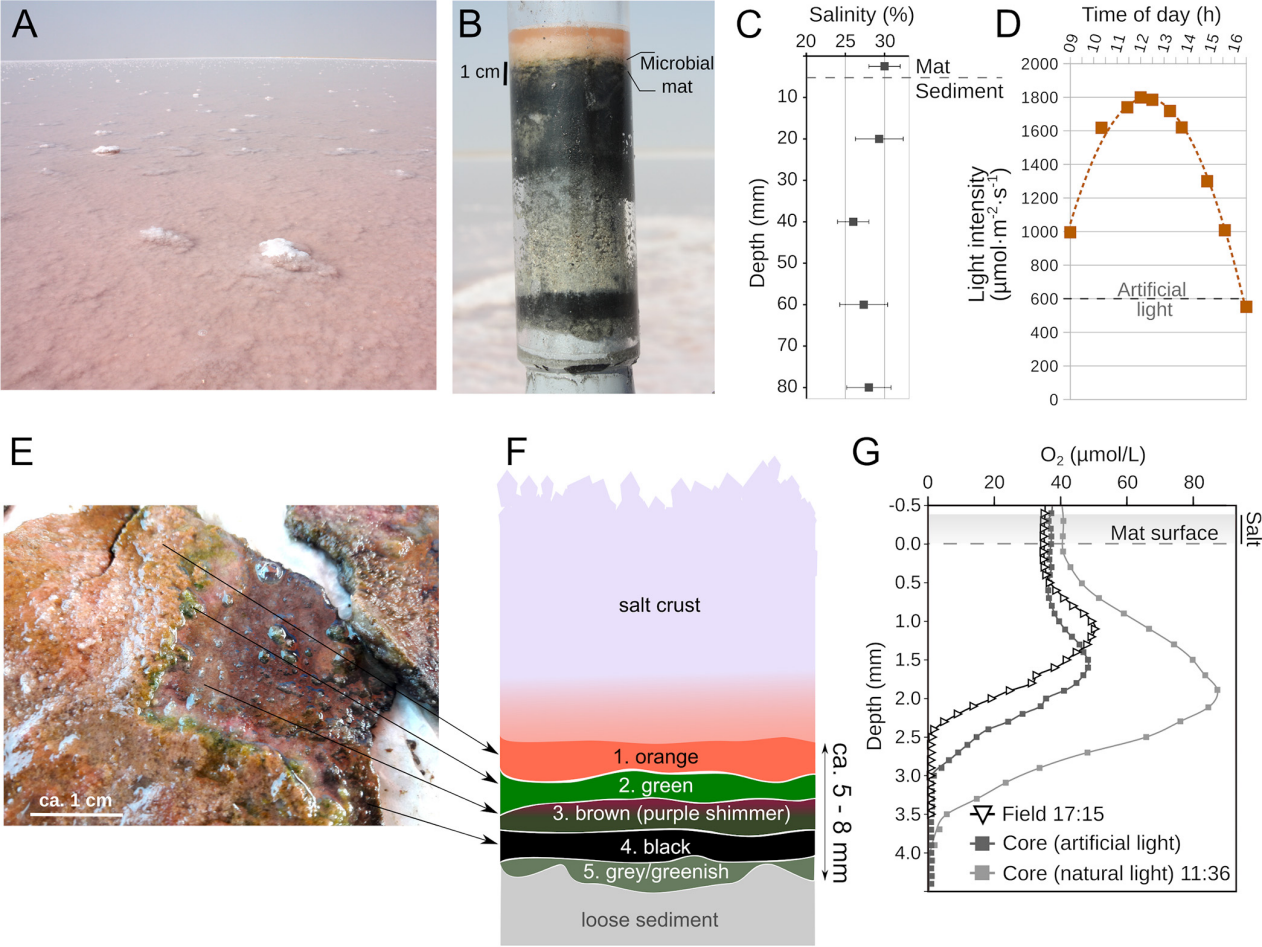
Overview of the sabkha near Shannah, Oman, and the salt covered microbial mats in February 2018. (A) Image of the sampling site in the salt-crust-covered tidal flat. (B) A core including sediment, microbial mat, salt crust, and salt-saturated water. (C) Depth profile of porewater salinity. Squares indicate average values from three cores, and error bars indicate standard deviation. (D) Light intensity measured over the course of a day with a PAR sensor. (E) A piece of microbial mat cleaned from the salt and with layers partially scraped off. (F) Scheme of mat layers based on observations of color and texture. (G) Representative microsensor profiles of O_2_ concentrations in the mats measured during the sampling campaign (at 30% salinity). Profiles were measured in the field and in sampled cores with artificial and natural illumination. Sunrise and sunset times are indicated to consider differences in light intensity.

The mats we studied were identified as a cohesive leathery structure easily separable from the sediment. The mats were laminated into five distinct layers, each 0.5 to 1.5 mm thick, and were (going from the surface downward) colored orange, green, brown, black, and gray (Fig. 1). No gypsum, calcium carbonate, or halite precipitates were found inside the mat matrices. The upper two layers (orange and green) were highly gelatinous while the lower two layers (black and gray) contained a higher fraction of sediment particles. The average porosity in the upper 5 cm was 0.35.

During the field trip in February 2018 (see Fig. S2 in the supplemental material), the temperature at midday below the salt crust was 35°C, which was 5°C above the air temperature. In the early morning, the temperature below the crust was 22°C, equal to the air temperature. The porewater salinity in the mats under the salt crust was 30% (Fig. 1). In January 2019, additional mat samples for laboratory experiments (Fig. S2) were collected from the same site at the same salinity and inundation conditions. At the Max Planck Institute (MPI) Bremen laboratory, measurements of chloride concentrations by ion chromatography revealed that the porewater salinity of salt-crust-covered mats without overlaying water was 40%. In order to simulate a tidal event, the cores were inundated in seawater (salinity, 3.5%). Three days after inundation, the porewater salinity in the mat dropped down to 12%. Below the mat, the salinity gradually increased with depth up to 30% at 3 cm. After the inundation experiment, the water covering the mats was left to evaporate (Fig. S2). A new salt crust formed within 3 days, while the porewater salinity in the mat increased back to 40%. The sulfate concentration in the porewater from the upper 5 cm remained within the same range before (230 and 390 mM) and after inundation (170 to 370 mM).

**FIG 2:**
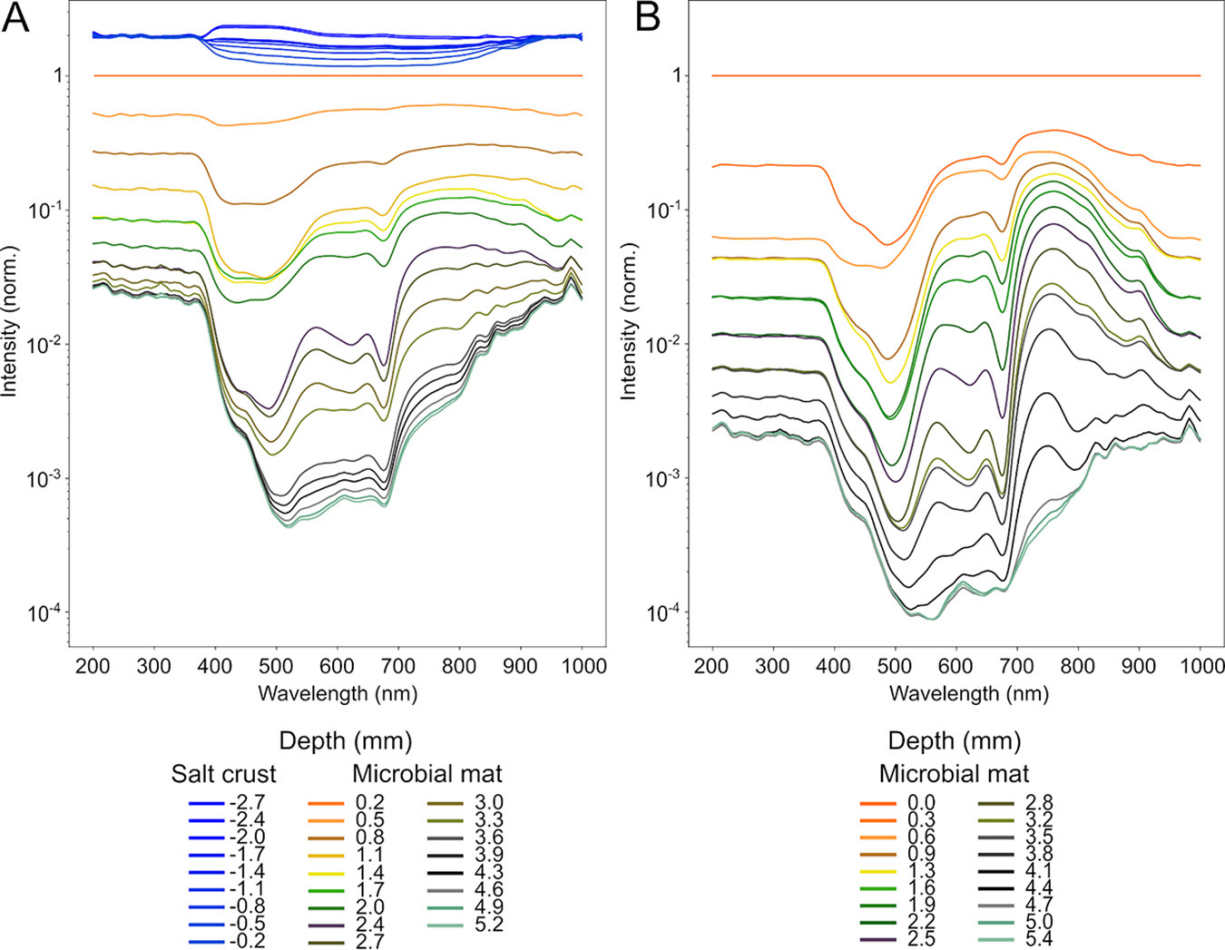
Spectral irradiance at different depths in the salt crusts and mats. Spectra in the mats with salt crust (A) and in mats after a simulated flood event (B). Note the low attenuation of light within the salt layers (blue traces at top) in panel A. The depth distribution of the irradiance spectra was normalized to the value at the mat surface. The depth layers are color-coded to approximately correspond to the determined mat layers (Fig. 1F) from orange at the mat surface to gray-green at the bottom of the mat. The spectral traces show the specific absorption of photopigments as well as the overall attenuation of irradiance by the sediment matrix.

We performed microscale spectral attenuation measurements to determine the light conditions beneath the crust. In the salt crust, rather little (∼1%) light attenuation occurred, and attenuation was rather uniform across the spectral range (Fig. 2A). This showed absence of light-harvesting pigments or sedimentary material in the salt layer. Below the salt layer, the light was attenuated exponentially as is typical for sedimentary matrices such as mats. When the salt crust was dissolved, a very similar pattern of spectra was observed (Fig. 2B). Within 3.5 mm into the mat (brown or black layer), photosynthetically active radiation (PAR) light was attenuated to 1% of the mat surface level both with and without the salt layer.

### Microbial processes.

We measured the essential metabolic processes, such as oxygenic and anoxygenic photosynthesis, respiration, sulfate reduction, and sulfide oxidation, under the salt crust at saturation-level salinity to test if the microbial community can remain metabolically active and sustain itself under this condition. These measurements were compared to measurements performed after inundation of the mats with seawater to mimic flood conditions.

### Oxygenic photosynthesis and respiration.

In *in situ* microbial mats and in freshly sampled cores (salinity, 30%), elevated oxygen (O_2_) concentrations were found in the upper 3 to 4 mm (corresponding to orange to brown layers) (Fig. 1), indicating oxygenic photosynthesis activity. However, when recreating field conditions at Sultan Qaboos University (SQU) in Muscat by inundating the cores with water from the site (salinity, 30%) for 3 days, no O_2_ production could be measured (see Fig. S3 in the supplemental material). Subsequent tide simulation by inundating the cores in 5% salinity water induced a high O_2_ peak in the mat after 2 days (Fig. S3). When the overlying water was then exchanged back to 30%, lower levels of O_2_ production could still be measured for up to 90 min after the change (Fig. S3).

**FIG 3:**
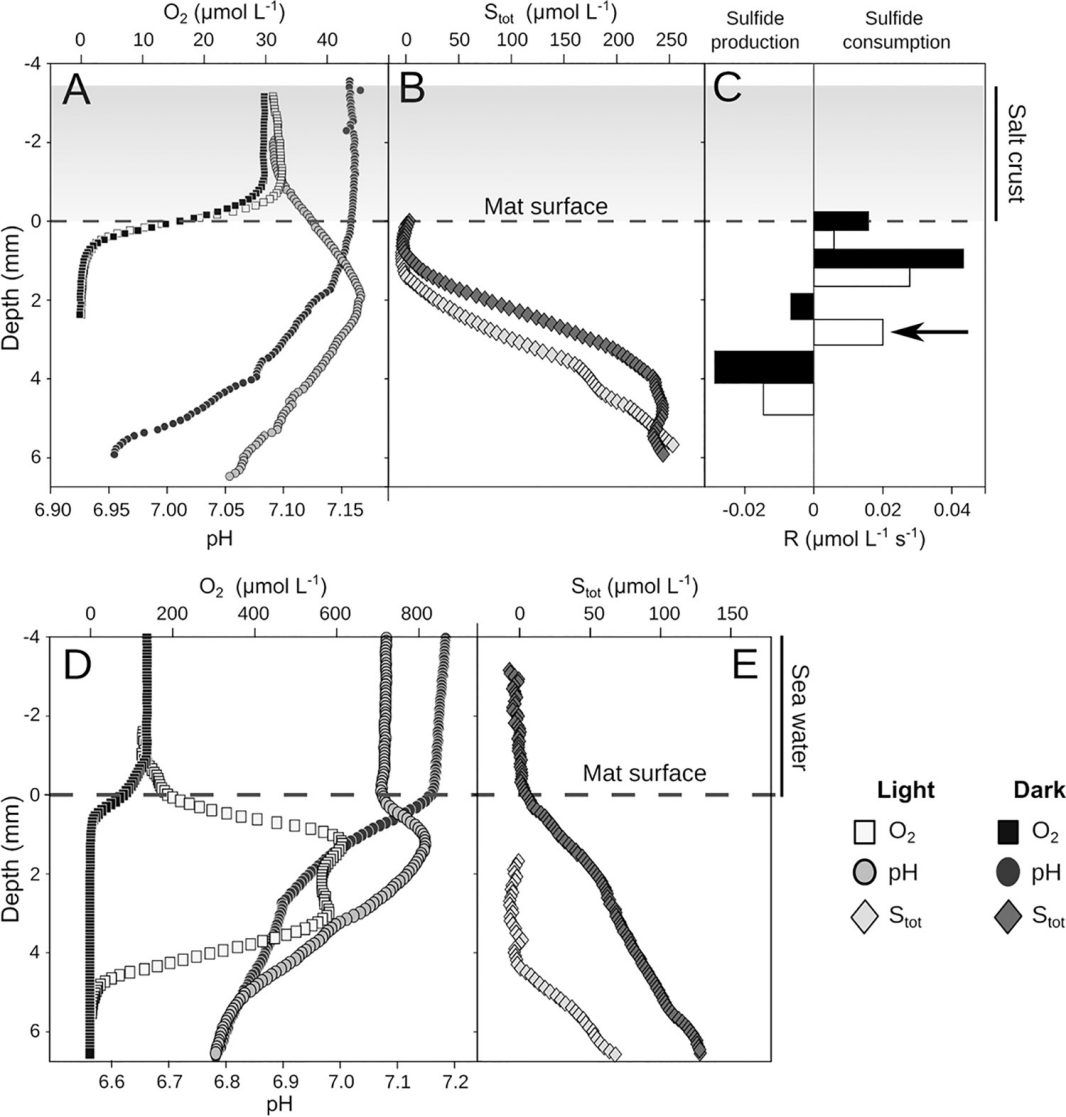
Depth profiles of O_2_, pH, and total sulfur (S_tot_) of mats sampled in January 2019. Representative steady state profiles of O_2_ (squares) and pH (circles) in a salt covered mat (A), representative steady state profiles of S_tot_ in a salt covered mat (B), local conversion rates of S_tot_ in a salt covered mat (arrow indicates sulfide consumption due to anoxygenic photosynthesis) (C), representative steady state profiles of O_2_ (squares) and pH (circles) 3 days after inundation with seawater (D), and profiles of S_tot_ 3 days after inundation with seawater (E). Note the order of magnitude difference in O_2_ concentrations between salt-covered (A) and flooded mats (D).

Further cores were transported to MPI Bremen for more detailed analysis. First, O_2_ profiles were measured in salt-crust-covered cores without overlaying water (porewater salinity was 40%, determined by ion chromatography chloride measurements). O_2_ profiles measured through the salt crust and into the mats did not show an O_2_ peak, and O_2_ levels did not change upon illumination, indicating complete absence of oxygenic photosynthesis (Fig. 3A). The O_2_ profile through the salt crust was flat, indicating absence of significant diffusion resistance. The cores were inundated in seawater and subjected to a 12-h light, 12-h darkness cycle, while O_2_ profiles were continuously measured (see Fig. S4 in the supplemental material). Two days after inundation by seawater and after the disappearance of salt crust, O_2_ production in light conditions commenced (Fig. S4) and increased further on the third day (salinity in the green layer dropped to 12%) (Fig. 3D). Two O_2_ peaks or shoulders developed, indicating two zones of photosynthesis, close to 2 and 4 mm below the mat surface (Fig. 3D). The respiration rate in the dark increased 9-fold upon decreased salinity (Table 1).

**TABLE 1:** Biogeochemical rates and fluxes^*a*^

Biogeochemical process	Under salt crust		Inundated mat	
	Areal (mol m^−2^ s^−1^)	Avg volumetric (mol m^−3^ s^−1^)	Areal (mol m^−2^ s^−1^)	Avg volumetric (mol m^−3^ s^−1^)
Oxygenic photosynthesis (O_2_ production)	0 ± 0	0 ± 0	Light, 9.0E−07 ± 3.0E−07	Light, 3.11E−04 ± 1.3E−04
Aerobic respiration	Dark, 2.1E−08 ± 0.4E−08; light, 2.4E−08 ± 0.1E−08	Dark, 1.3E−05 ± 0.4E−05; light, 1.24E−05 ± 0.3E−05	Dark, 1.9E−07 ± 0.05E−07; light, 4.7E−07 ± 1.4E−07	Dark, 1.1E−04 ± 0.3E−04; light, 1.55E−04 ± 0.45E−04
Sulfate reduction	Over 3 cm, 3.4E−09 ± 0.6E−09	Mat, 6.2E−08 ± 3.9E−08; avg 3 cm, 1.2E−07 ± 0.7E−07	Over 3 cm, 6.7E−08 ± 2.6E−08	Mat, 1.1E−06 ± 6.2E−10; avg 3 cm, 2.4E−06 ± 3.3E−06
Sulfide flux	Dark, 8.4E−08 ± 1.2E−08; light, 7.6E−08 ± 0.6E−08		Dark, 2.1E−08 ± 0.1E−08; light, 3.2E−08 ± 0.4E−08	

a Rates and fluxes are calculated from three steady state microprofiles as described in reference 71, except for SO_4_^2−^ reduction rates calculated based on ^35^S measurements. Volumetric respiration and photosynthesis rates represent average values across mat depth where O_2_ concentrations were above zero.

### Sulfide oxidation and anoxygenic photosynthesis.

The sulfide and O_2_-profiles (Fig. 3) showed an overlap of the sulfidic and the oxic zone, where sulfide was consumed aerobically. The pH under the salt layer in the dark decreased with depth but showed a small peak at a 2-mm depth (green layer) under (artificial) illumination (Fig. 3A). Sulfide diffused upward from a 5-mm depth and, under illumination, receded slightly and decreased in concentration (Fig. 3B). The pH peak and the sulfide recession during illumination indicated anoxygenic photosynthesis activity below 2-mm depth. The areal sulfide fluxes, where overlapping with the oxic zone, were similar in light and dark. Although from such measurements no areal rates of anoxygenic photosynthesis could be calculated, the minimum distribution of net anoxygenic photosynthesis could be confined to between 2 and 4 mm from the mat surface as the zone of net sulfide consumption under illumination (Fig. 3C).

### Sulfate reduction.

**FIG 4:**
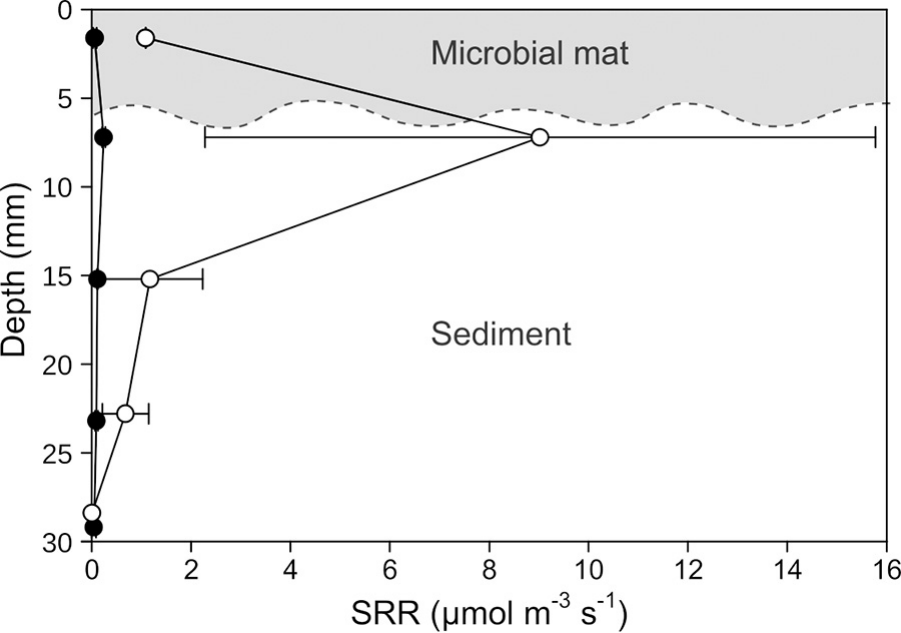
Sulfate reduction rates (SRR), determined by the ^35^S method, in mats covered by a salt crust (black circles) and in mats after a simulated flood event (white circles). The dashed line indicates an approximate delineation between mat and sediment. Considering the slightly varying mat thickness, the peak of sulfate reduction might lie in the lowest mat layer or in the sediment directly underneath the mat.

Sulfate reduction in the mat and underlying sediment (up to a 30-mm depth) was detected under the salt crust (40% salinity) and in mimicked flood conditions (12% salinity); several samples were below the detection limit of 0.05 μmol m^−3^ s^−1^, and the rates were rather variable between cores. Under flood conditions, average sulfate reduction rates in the mat increased 18-fold (Table 1). However, the highest rates were found at a 7-mm depth (Fig. 4), which, considering not perfectly even mat thickness, could be in the gray mat layer or the sediment directly below the mat. Interestingly, the sulfide fluxes decreased upon reduction of the salinity, indicating a rebalancing between production and consumption processes (an increase in sulfide oxidation).

### Microbial community stratification.

We determined the fine-scale stratification of the microbial community, catalyzing the measured processes based on distribution of photopigments correlated to microscopic observation and quantification of microbial cells. The taxonomic identities of microbial community members in different layers were determined by amplicon sequencing of the 16S rRNA gene.

### Distribution of phototrophs based on photopigment stratification.

**FIG 5:**
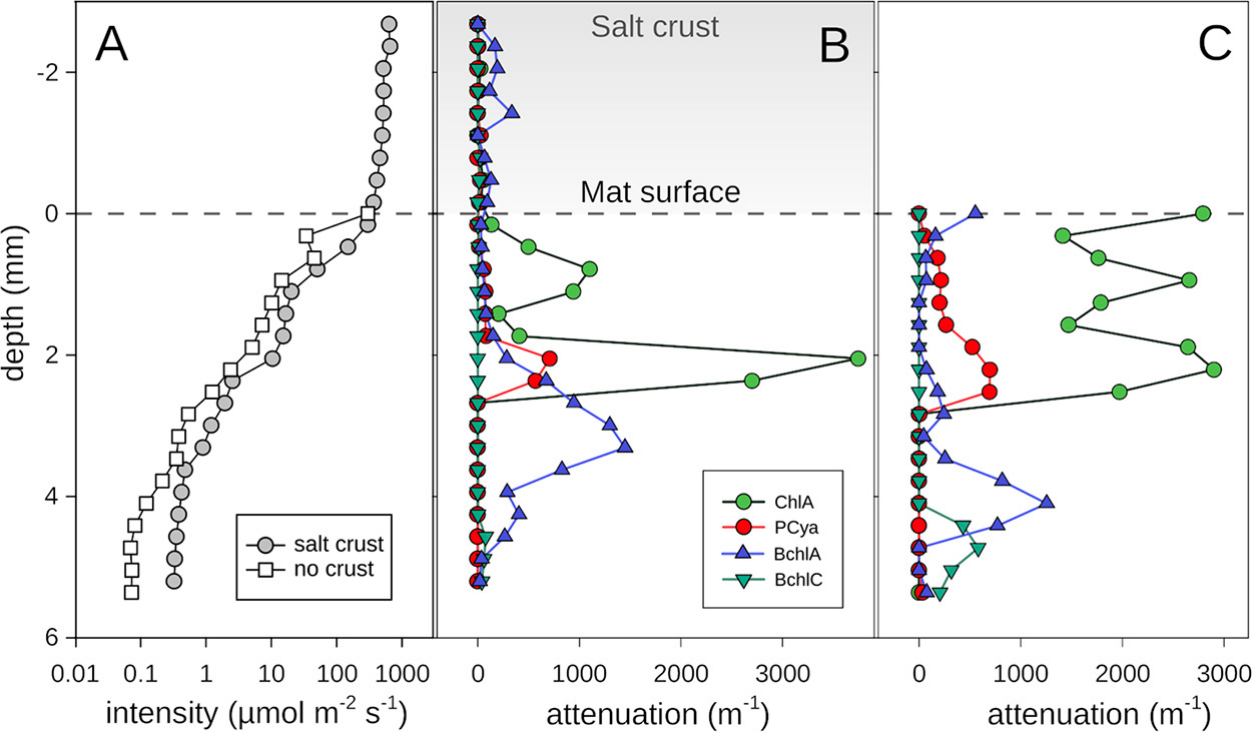
Photosynthetically active radiation intensity profile and photopigment abundances estimated from absorbance peaks. (A) Integrated photosynthetically active radiation intensity profile within the salt crust and the mat. Photopigment abundances estimated from the absorbance peaks in the attenuation spectra in the salt crust and mats underneath (B) and in mats after a simulated flood event (C). Pigment abbreviations in panels B and C are as follows: ChlA, chlorophyll *a*; Pcya, phycocyanin; BchlA, bacteriochlorophyll *a*; BchlC, bacteriochlorophyll *c*.

**FIG 6:**
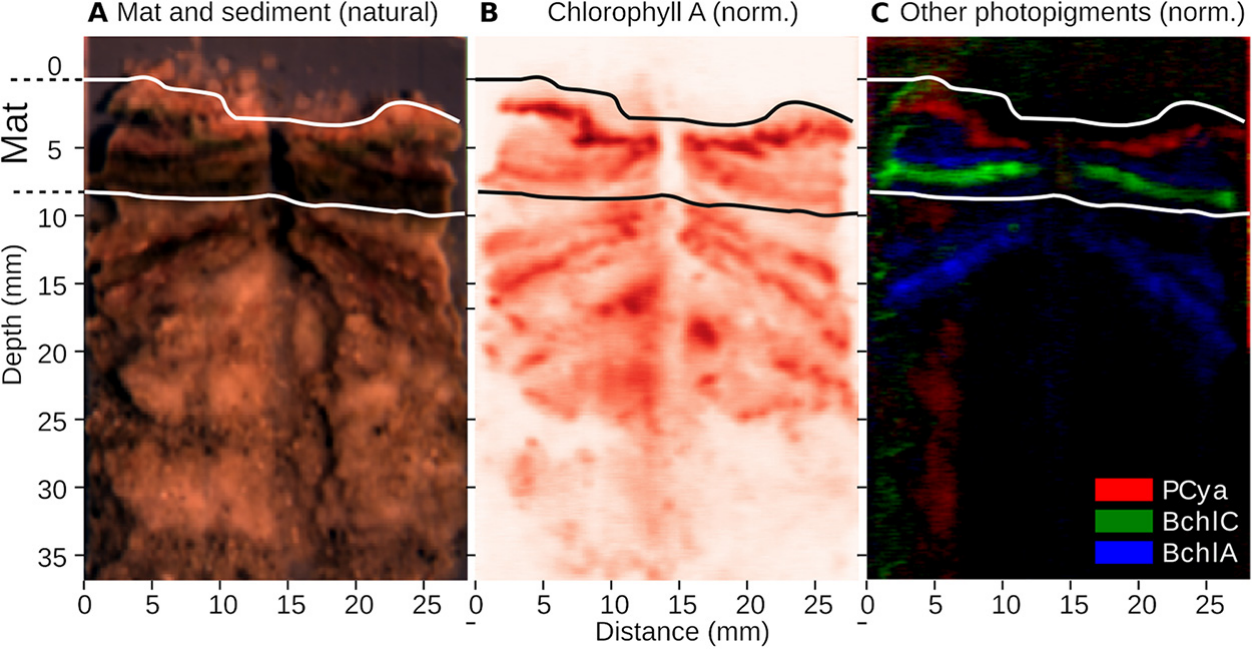
The distribution of photopigments across the cross-section of a core with the mat and underlying sediment. Photopigment distribution was assessed using hyperspectral imaging. The upper and lower limits of the mat are indicated by horizontal lines. Note that thickness varies between 5 and 8 mm. (A) Natural color-rendering derived from the rectified hyperspectral image. (B) Abundance estimates of chlorophyllic pigments based on spectral derivative analysis as a false-color map. Chlorophyllic pigments detected below the mat likely represent debris of phototrophic microorganisms, as supported by presence of chlorophyll degradation products (see Fig. S5 in the supplemental material), absence of phycocyanin (C), and phototrophic cells (see Fig. S6 in the supplemental material) in lower mat samples, as well as no light penetrating to these depths (Fig. 5A). (C) Group-specific pigments phycocyanin (*Cyanobacteria*) and bacteriochlorophylls *a* (purple bacteria, e.g., *Rhodovibrio*) and *c* (*Chloroflexi*) as composite color map.

The distribution of photopigments, as assessed by microscale spectral attenuation (Fig. 5) and hyperspectral imaging (HSI) (Fig. 6), showed absorption signals corresponding to chlorophyll *a* (Chl-*a*) (675 nm), phycocyanin (625 nm), bacteriochlorophyll *a* (BChl-*a*) (845 nm, 902 nm), and bacteriochlorophyll *c* (BChl-*c*) (745 nm). The depth distribution of Chl-*a* determined by spectral attenuation showed two peaks separated by ∼1 mm (Fig. 5B). With HSI, the two peaks were less distinguishable and, based on depth, likely appear as one main upper layer (Fig. 6). The depths of the Chl-*a* bands determined from spectral attenuation profiles (Fig. 5B and C) differed slightly from the depths determined by HSI, probably due to lateral variability in the structure of the mats, which is well depicted in the hyperspectral images (Fig. 6). Additionally, HSI revealed multiple weak layers of chlorophyllic pigments and derivatives in the black and gray layers of the mat and in the sediment below. These lower chlorophyllic layers seen in HSI were also observed in chromatographic analysis of pigments extracted from 2018 mat samples (see Fig. S5 in the supplemental material). In chromatographic analysis, Chl-*a* was present throughout the profile, peaking in the green layer (∼1- to 2-mm depth) and showing a second, lower peak in the gray layer (∼4 to 5 mm). However, chromatography showed that the gray mat layer also contained increased proportions of chlorophyll degradation products, such as chlorophyllide *a* and pheophorbide *a*. Together with the microscopic observation of empty cyanobacterial sheaths at this depth (see Fig. S6 in the supplemental material), it suggests that chlorophyll pigments are not functional in these layers.

A layer of phycocyanin, a water-soluble antenna pigment produced by cyanobacteria, was correlated to the main Chl-*a* layer (Fig. 5 and 6), with spectral attenuation measurements locating it at the depth of the lower Chl-*a* peak (Fig. 5). BChl-*a* was detected below the main Chl-*a* layer and above the BChl*-c* layer with both methods (Fig. 5 and 6). BChl-*a* was peaking at 3 to 4 mm and BChl*-c* below 4 mm. The depth distributions of pigments were similar before and after removal of the salt layer by inundation, showing that there was no dramatic change of distribution of phototrophs due to change in salinity (Fig. 5B and C). However, some minor differences were observed after the salt dilution, such as an increase of Chl-*a* and BChl-*a* at the very surface, a minor increase in phycocyanin in the upper 1.5 mm, and appearance of BChl*-c* peak at a 5-mm depth.

### Distribution of phototrophs by microscopy.

Cell numbers per gram mat wet weight were determined by fluorescence microscopy of fixed material (see Fig. S6 and S7 in the supplemental material). The cell numbers in the upper three layers of the mat (orange, green, and brown, 1.2 × 10^10^ to 1.6 × 10^10^) were four times higher than in the lower layers (black and gray, 3.5 × 10^9^ to 4.3 × 10^9^) (Fig. S7). *Cyanobacteria* and *Chloroflexi* were distinguished by their morphology and autofluorescence (Fig. S6). *Cyanobacteria* cells were large in size but low in number (Fig. S6 and S7). The highest number and proportion of filamentous *Cyanobacteria* cells were observed in the green layer (1 × 10^9^). The highest numbers of large unicellular *Cyanobacteria* were observed in the brown layer (1 × 10^8^). *Chloroflexi* had the highest numbers in the green (2.2 × 10^9^) and brown (2.9 × 10^9^) layers.

Besides lower cell numbers, the black and gray layers also contained a larger amount of organic debris, such as empty cyanobacterial sheaths and possibly aggregates of dead microbial cells, visible as a faint fluorescence in the 4′,6-diamidine-2′-phenylindole dihydrochloride (DAPI) channel (Fig. S6). The cells in the lower two layers were visibly thinner than in the upper three layers.

### Stratification of microbial community by 16S rRNA gene analysis.

**FIG 7:**
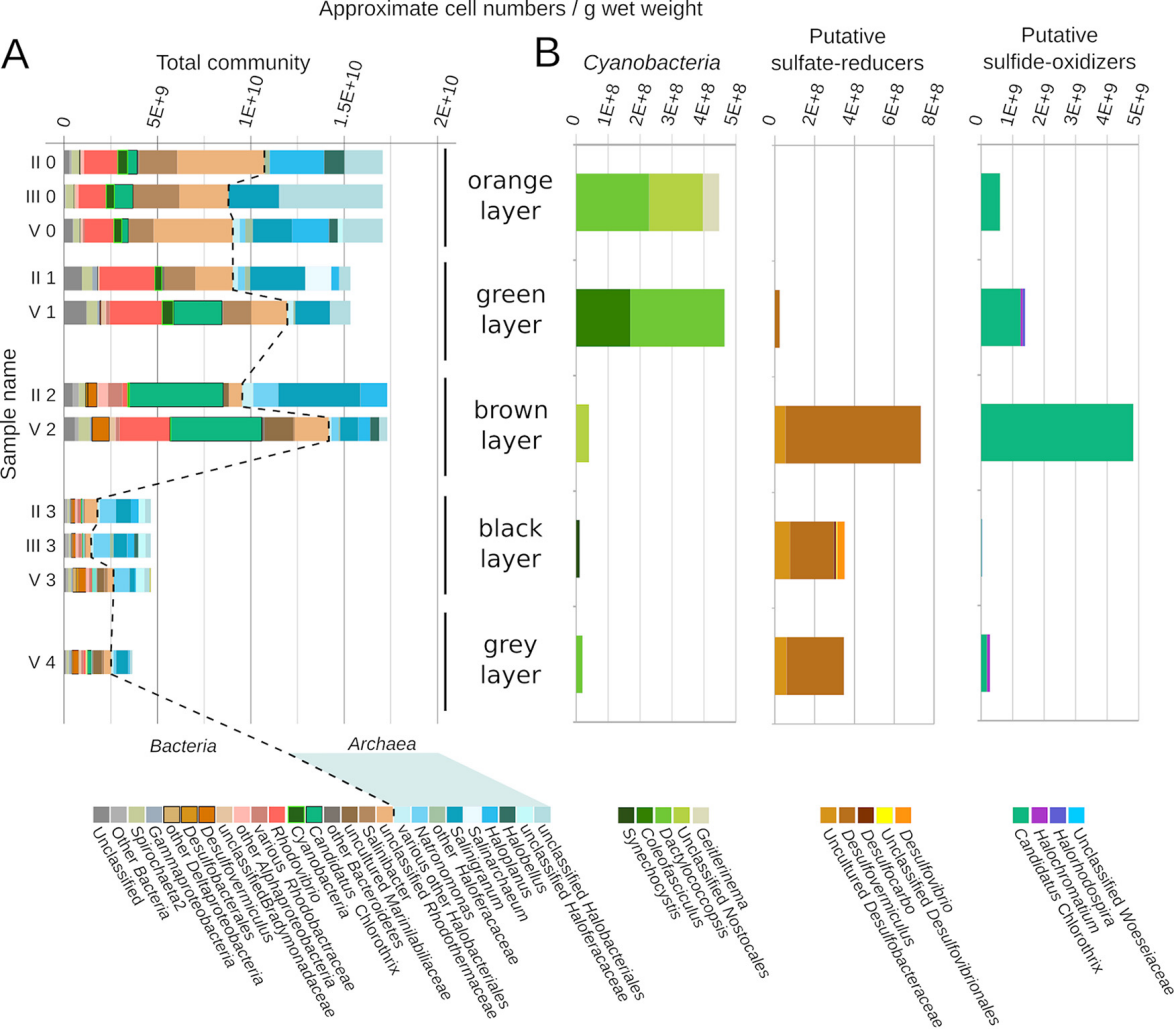
Microbial community composition as determined by 16S rRNA gene amplicon sequencing. The relative amplicon sequence abundances were multiplied by total cell counts per gram sediment obtained via fluorescence microscopy (see Fig. S7 in the supplemental material) as follows: (Cell number/g sediment)_taxon_ = (Relative sequence abundance)_taxon_ × (Total cell numbers/g sediment). (A) The composition of the total microbial community shown for two to three replicates per layer (exception layer 4 to 5 mm). In the sample name, the roman number indicates the mat patch replicate and the arabic number indicated the layer from “0” being the surface orange layer to “4” being the bottom gray layer. (B) Estimated cell numbers of photoautotrophs (*Cyanobacteria*), sulfate-reducing bacteria (all *Deltaproteobacteria*), and sulfur-oxidizing bacteria in each layer. Full data on relative sequence abundances is provided in File S1 in the supplemental material.

The sequence analysis supported a community that was clearly structured in layers containing different phototrophs (Fig. 7; see also File S1 in the supplemental material). Two groups of primary producers were found—oxygenic phototrophs and potential anoxygenic phototrophs. Remarkably, *Cyanobacteria* constituted a relatively minor proportion of the sequence reads (Fig. 7A). Cyanobacterial sequences in the uppermost orange layer harbored unclassified *Nostocales* (1%), *Dactylococcopsis* (1.4%), and *Geitlerinema* (0.4%) (Fig. 7B), while the green layer contained *Dactylococcopsis-* (2%) and *Coleofasciculus*-related (1.1%) taxa. In the brown layer, only unclassified *Nostocales* (0.24%) were detected. Sequences related to *Synechocystis* were only found in the black layer (0.24%).

Regarding anoxygenic phototrophs, the brown layer was dominated by sequences classified as “*Candidatus* Chlorothrix” (29%) (Fig. 7A), an anoxygenic sulfur-oxidizing phototroph from the *Chloroflexi* phylum (31). Other known sulfur-oxidizing taxa, be it phototrophic or chemolithotrophic, were barely present (Fig. 7B). Thus, sulfide oxidation is probably strongly dominated by anoxygenic phototrophic *Chloroflexi*.

Sequences attributed to sulfate-reducing bacteria (SRB) (Fig. 7B) were found mainly in brown, black, and gray layers and were dominated by *Desulfovermiculus*. SRB were clearly excluded from the oxic zone.

Large fractions of the sequences were assigned to heterotrophic or photoheterotrophic microbial clades, neither contributing to primary production nor involved in the S cycle, such as photoheterotrophic *Rhodovibrio* that were abundant in the upper layers (maximum 19% in the green layer). Other highly abundant groups were *Salinibacter* (10% in the orange and green layer) and an unclassified genus of *Rhodothermaceae* (23% and 13% in the orange and green layer, respectively). The lower three layers showed increased proportions of uncultured *Marinilabiliaceae*, likely anaerobic fermenters (13% in the black layer).

We noticed high relative abundances of archaeal sequences within the mats, reaching a maximum in the black layer. In this layer, archaeal sequences constituted, on average, 56% of all reads, with *Halobacteriales* genera *Salinigranum*, *Haloplanus*, and *Natronomonas* being the most abundant (Fig. 7A). Among archaeal sequences, we detected only two potentially methanogenic taxa, not further classified members of *Methanosarcinaceae* and *Methanofastidiosales*, whose relative abundance did not exceed 0.03% in any of the samples.

## DISCUSSION

Investigation of microbial activity at various salinities, ranging from 12% (wt/vol) simulating a flood, 30% in the field, and 40% in mats with fully evaporated overlaying water, revealed that metabolic processes were differently susceptible to salinity stress. At saturation salinity (40%), we were able to measure anoxygenic photosynthesis, aerobic respiration, phototrophic sulfide oxidation, and some sulfate reduction, which is largely in agreement with current knowledge on microbial communities in salt-saturated environments (15). Oxygenic photosynthesis was shown to be more restricted and could only be detected up to a salinity of 30%. Regarding the salt crust, we could not confirm any significant protection against dehydration and irradiation for the microorganisms in the underlying microbial mat. The microprofiles showed that the salt layer forms no resistance to O_2_ transport, and thus we conclude that gases, including water vapor, can easily exchange, and the mats below the salt remain sensitive to desiccation. The temperature below the crust at midday was 5°C higher, while the seawater surface temperature was 9°C higher than air. Therefore, it seems that the salt crust does reduce the mat temperature to some extent as also noticed by Baas Becking (28). Our data further show that the salt transmitted PAR easily (Fig. 5A), and also UV and near-infrared radiation were hardly attenuated (Fig. 2). The idea that microbes below such a salt layer “are, in terms of light intensity, practically living in the deep sea” (28) thus cannot be supported. Instead, light can easily penetrate salt crusts, allowing phototrophy in the underlying mat if the microbial community can perform it under salt-saturated conditions.

### Primary production.

Our results clearly show the absence of oxygenic photosynthesis and cyanobacterial primary production at saturation-level salinities (40%). At salinities of around 30%, the results seem more ambiguous at first. In 2018, we were able to measure O_2_ oversaturation at 30% salinity *in situ* and in the field laboratory (Fig. 1). In 2019 however, after transport to the laboratory at SQU, O_2_ production in the mat at 30% was only measurable after first inundating the mat with low-salinity water (5%) and then returning back to 30% (see Fig. S1 and S2 in the supplemental material). It is important to note that these salinities (5% and 30%) are salinities of the overlaying water, not of the porewater within the mat, which might take longer to equilibrate. This suggests that the upper salinity limit for oxygenic photosynthesis in these mats lies somewhere around 30% NaCl (wt/vol). Oxygen production at such high salt concentrations has not been measured previously. *Dunaliella* algae are assumed to be the sole primary producer in salt-saturated brines based on their high abundance (17) and unpublished reports on photosynthesis in these environments (32). In culture, *Dunaliella* photosynthesis has only been measured up to a salinity of ca. 18% (3 M NaCl) (21–23), while growth is known to occur up to 29% (5 M NaCl) (24, 25), albeit at much lower rates. Unicellular *Cyanobacteria* were previously shown to grow in special media saturated with NaCl but not in salt-saturated natural waters (33).

Although *in situ* O_2_ production by unicellular cyanobacteria has been indicated in halite crystals from the Atacama Desert (13), the very high reported O_2_ concentrations even in the dark (4 to 5 mg/liter, exceeding the O_2_ solubility of 2.8 mg/liter at 20°C, 20% NaCl) suggest that the salinity of the medium was in fact below saturation (based on O_2_ concentrations in the dark, probably 10 to 15% NaCl). The same study also reported pulse-amplitude modulated fluorescence measurements indicating activity of photosystem II (13). However, this method is challenging to apply to cyanobacteria, where it might measure fluorescence of phycobilisome pigments instead of photosystem II (34, 35). Further, presence of functional and active chlorophyll does not necessarily indicate ongoing oxygenic photosynthesis. Phototrophic microorganisms can use their photosystems to generate proton-motive force and ATP via cyclic electron transfer without coupling to CO_2_ fixation (36). Such photoheterotrophic mode of operation is also suggested to play a role in desiccation survival by arid surface soils microorganisms (37). Studies monitoring *in situ* O_2_ production in hypersaline microbial mats up to 26% NaCl showed a sharp decline of photosynthesis with increasing salinity (26, 27, 38). Extrapolating these data also suggested an upper salinity limit for oxygenic photosynthesis of 30% (26), which is supported by our experimental data. During our fieldwork, the mats were covered with water indicating a recent influx of low-salinity water, and that *in situ* salinity was below saturation. In the absence of dilute water, the salinity will rapidly increase to saturation, as it happened within 3 days in our laboratory experiment even at room temperature and under artificial light. A transect study of the tidal flat found the same area dry in December 2014 (30), indicating fluctuating water levels. Considering higher temperatures and irradiation in summer and lack of daily tides at the site, the mats will often be exposed to saturation-level salinities under which their photosynthetic activity is halted.

Anoxygenic photosynthesis, conversely, was detected even at salt saturation conditions, based on reduced sulfide concentrations under illumination (Fig. 3). This process is thus more robust against salinity stress, allowing the anoxygenic phototrophs like “*Candidatus* Chlorothrix” (31) to prosper. Also, some of the *Cyanobacteria* might be capable of anoxygenic photosynthesis (39, 40). Since phototrophic sulfide oxidizers do not produce O_2_, they are not limited by decreased O_2_ solubility at high salinities as suggested for oxygenic phototrophs (26).

### Oxygenic phototrophs.

In agreement with a previous study (30), the mat community contained several taxa of *Cyanobacteria*, including filamentous genera like *Coleofasciculus* and *Geitlerinema*, which is unusual for such hypersaline environment (16). While low relative abundances of *Cyanobacteria* in sequencing data are known from other hypersaline mats (1, 2), they may be an underestimation of cyanobacterial biomass as indicated by our fluorescence microscopy data (see Fig. S4 and S5 in the supplemental material) and supported by pigment analysis. The combination of optical profiling, hyperspectral scanning, microscopy, and 16S rRNA-based analysis provide a coherent picture of a stratified and complex phototrophic community consisting of two layers of different *Cyanobacteria*, below which a zone of anoxygenic phototrophs resides. The double peak in O_2_ production aligns with these observations. The spatial separation of two different groups of cyanobacteria could be explained by adaptations to different microenvironments. These microenvironments, ca. 2 mm apart, differed in several parameters as follows: within 2 mm, the light levels attenuated with 1 to 2 orders of magnitude (Fig. 5A); salinity was likely lower in the upper photosynthetic zone during inundation; and during illumination, sulfide never reached the upper zone. Sulfide is a toxic compound that can inhibit photosynthesis in many cyanobacteria, while other species are resistant to sulfide or are even stimulated by it (41). Possibly, the mostly unicellular *Cyanobacteria* in the upper zone are more sensitive to sulfide, whereas the *Coleofasciculus* in the lower layer are more sulfide tolerant. *Coleofasciculus chtonoplastes* (previously *Microcoleus chtonoplastes*), for example, was shown to be capable of phototrophic sulfide oxidation (42). Unicellular *Dactylococcopsis* seems to have a broad sulfide tolerance range, as its sequences were present in both layers. Species of nonmotile *Synechocystis*, detected in the brown layer, were reported to contain genes linked to sulfide oxidation, and it is speculated that they can perform anoxygenic phototrophy on sulfide (43).

In the inundation experiment where high photosynthesis rates were observed, the overall amount of photopigments did not increase strongly (Fig. 5), indicating that the increase in activity was due to a revived community and not due to an increase in phototrophic population or reconstitution of structurally altered pigments (44, 45). This similarity in vertical structure and population size of the phototrophic community in the mat with salt crust and the inundated mat indicated that salinity change induced only minor migration compared to previously studied hypersaline (46, 47) and desiccated (45) microbial mats and likely no substantial growth of the phototrophs.

### Anoxygenic phototrophs.

Similar to other hypersaline microbial mats (2), we observed a large fraction of *Chloroflexi* sequences in the brown layer below the cyanobacteria-containing layers, which overlaps well with the layering of Chl-*a* and BChl*-c* as determined from the light spectra (Fig. 5) and HSI (Fig. 6). The activity of *Chloroflexi* is hard to assess due to their large metabolic flexibility. They can act as heterotrophic and autotrophic anoxygenic phototrophs and can respire a large diversity of compounds with O_2_, including sulfide (48–52). The detected *Chloroflexi* (closely related to “*Candidatus* Chlorothrix halophila”) probably dominated the sulfide oxidation, as other potentially sulfur-oxidizing taxa were barely present in the sequence data. BChl-*a*, which is usually assigned to purple sulfur bacteria, probably stems from photoheterotrophic *Rhodovibrio* (53), whose sequences have high relative abundances in the orange, green, and brown layers. However, it is noteworthy that the peak of BChl-*a* was observed below the cyanobacteria layers, whereas *Rhodovibrio* sequences are also abundant at the very top of the mat. This could mean that the *Rhodovibrio* populations in the orange and green layers rely entirely on organoheterotrophic energy generation, whereas the population deeper in the mat produces bacteriochlorophyll for light harvesting.

### Sulfate reduction.

Sulfate reduction rates under the salt crust at a salinity of 40% were low but detectable. However, upon decreasing the salinity, sulfate reduction rates in the mat increased 18-fold (Fig. 4; Table 1). While originally thought to be thermodynamically unfavorable at high salinities (54), some studies have reported low, yet present, sulfate reduction rates in hypersaline sediments and mats (11, 55–57). Other recent reports, however, still come to the conclusion that sulfate reduction is inhibited at salt saturation (58, 59). Our data suggest that at extreme salinities, even if growth of sulfate reducers is stopped, energy generation might still continue, albeit at a lower rate. Unlike in other mats, where SRB and significant sulfate reduction rates were found in oxic layers (60–63), the relative abundance of SRB increased with depth following the classic concept of anaerobic organisms being confined to anaerobic zones. Sulfate reduction in the photic zone remained low even after reducing the salinity. The highest sulfate reduction rates were measured at a 7-mm depth, which is at the lower border of the mat or even below the mat itself. Thus, we conclude that the main source of sulfide is the underlying sediment.

Although the sulfur cycling is profoundly influenced by the salinity, using microsensors, we found similar sulfide profiles and total sulfide pools in mats at high and low salinities. With a total sulfide pool of 0.2 mol m^−3^ (Fig. 3) and a sulfate reduction rate of 2.4 × 10^−6^ mol m^−3^ s^−1^ (Table 1), the turnover rate of sulfide is approximately 1 day during low-salinity periods. Thus, as the sulfate reduction increases with a factor of 20, the sulfide consumption must also increase by the same factor, as otherwise the total sulfide pool would increase with 0.2 mol m^−3^ each day. As sulfide oxidation occurs mainly by anoxygenic photosynthesis, this process must also be strongly stimulated by reducing the salinity.

### Note on heterotrophic microorganisms and archaea.

Besides microorganisms participating in CO_2_ fixation and sulfur cycling, we detected a variety of heterotrophic or photoheterotrophic microorganisms, such as various *Halobacteriales* archaea, *Rhodovibrio*, and *Salinibacter* (53, 64, 65), which were previously mostly known to have planktonic lifestyles. Considering the high relative abundance of their sequence reads in the mats, these microorganisms likely have an active role in the mat community and contribute to carbon cycling. The proportion of *Archaea*, represented by members of known mostly photoheterotrophic taxa (various members of *Halobacteriales*), was much higher than the usually assumed 1 to 20% in mats (1). In fact, with 56% of archaeal sequences in the black layer, this is so far the highest reported proportion for microbial mats (56). Although it has been observed that methanogens can outcompete or live syntrophically with sulfate-reducing bacteria in hypersaline mats (66), this seems not to be the case in the Oman mats, as sequences attributed to methanogenic archaea were barely present. However, we cannot exclude the possibility that the methanogenic zone in this environment lies deeper in the sediment.

### Conclusion.

Although the salt crusts offer little protection in terms of attenuating light or maintaining humidity, the underlying microbial mat community retained the capability to perform all essential tasks for a viable ecosystem with internal recycling of the main elements as follows: primary production and mineralization of organic matter. However, this theoretical capability is not always operational, as oxygenic photosynthesis activity is inhibited during the saturation salinity (40%) periods. As no daily tides were observed at the site, these salinities are likely to occur during summer, whereas inundation occurs either during landward storms or winter rains. At saturation salinities, primary production can be sustained for a while by anoxygenic phototrophy; however, the reducing power needed for CO_2_ fixation is provided by sulfide, which is in turn supplied by sulfate reduction fueled by organics. As losses of reduced matter will inevitably occur (organics or sulfide), an ecosystem reliant on anoxygenic phototrophy for primary production and sulfate reduction for mineralization cannot be sustained. The microbial ecosystems below salt crusts, thus, ultimately depend on periods of reduced salinity allowing oxygenic photosynthesis or on import of reduced substances, e.g., from other ecosystems or from geothermal venting. Additionally, strongly reduced rates of other microbial energy generation processes, such as aerobic respiration and sulfate reduction, suggest that the microorganisms in the studied mats are adapted to endure and survive the saturated salinity conditions, if sporadically diluted, rather than to thrive in perennially saturated conditions.

## MATERIALS AND METHODS

An overview chart of the sampling events and order of conducted experiments can be found in Fig. S2 in the supplemental material.

### Site description and sampling.

Microbial mats were sampled at an irregularly inundated tidal flat in Oman (20°45'39.6"N 58°38'52.3"E), approximately 6.5 km from the low water line in February 2018 (Fig. 1; see also Fig. S1). During our sampling, the area up to 5 km away from the coast was affected by daily tides, whereas stagnant water (30% salinity) and halite crust (composition in Table S1 in the supplemental material) covering the bottom was observed further inland (Fig. S1). The whole area is covered by microbial mats with their appearance, thickness, and composition changing with the distance to the sea (as described in reference 30). These mats are naturally exposed to drastic salinity changes. When water evaporates, salinity approaches saturation and accumulating marine salts form a crystalline crust on top of the mats (Fig. 1). Tides, conversely, rapidly bring in low-salinity water (ca. 5%), dissolving the crust. For this study, we sampled microbial mats at the upper tidal line that were covered by several millimeters of crystalline salt and ca. 4 cm of stagnant water. The mats, coherent leathery biofilms, grew on top of permeable sandy sediment and were covered by a 2- to 3-mm-thick salt crust. Mats were easily liftable off the sediment with only a thin layer of loose sediment grains sticking to the downside of the mat. Mats were recovered in February 2018 and January 2019; an overview of conducted measurements is provided in Fig. S2 in the supplemental material.

Light intensity reached 1,799 μmol · m^−2^ · s^−1^ at 12:00 and fell to 552 μmol · m^−2^ · s^−1^ at 16:30 (Fig. 1D). On site, salinity of the overlaying water (measured throughout the sampling campaign) and porewater extracted from three sediment cores with Rhizons (Rhizosphere Research Products, Wageningen, The Netherlands), hydrophilic porous (pore size, 0.15 μm) polymer tubes connected to a syringe by a polyvinyl chloride tube (67), was measured with a refractometer after a 1:10 dilution. Crystalline salt was collected and sent for ion composition analysis to the Austrian Agency for Health and Food Safety (AGES) (Table S1).

Sediment sampling for porosity determination was conducted in 5-cm intervals from 0 to 20 cm depth from three different cores. Volumes of 2.5 ml of sediment were collected using cutoff syringes and transferred to exetainers. Porosity was determined by drying a known volume of sample at 60°C to a constant weight.

Three mat patches were collected approximately 1 m away from the O_2_ measurement site for DNA, pigment, and microscopic analysis. For these samples, the salt crust was removed from the mat and remaining salt crystals washed off with deionized water. For each of the three mat patches, the layers were separated with a scalpel according to their color—orange, green, brown, black, and gray (each 0.5 to 1.5 mm thick) (Fig. 1)—and stored in separate tubes at −20°C. A fraction of the material was preserved for fluorescence microscopy analysis; it was fixed with 4% paraformaldehyde (PFA) in 1× phosphate-buffered saline (PBS) (pH = 7.6) for 1.5 h at room temperature, washed twice with 1× PBS, and stored in an ethanol/PBS mixture (1:1) at −20°C until further analysis. A second fraction was stored in separate tubes at −20°C until pigment analysis.

Cores from the same site were collected in January 2019 at exactly the same salinity and flooding conditions (30% salinity, 2- to 4-cm water level) and brought to the laboratories of Sultan Qaboos University (SQU) in Muscat, Oman, and to the Max Planck Institute (MPI) in Bremen, Germany, for detailed biogeochemical analyses (Fig. S2). Cores were transported without the overlaying water (but with the salt crust covering the mat) to avoid accumulation of sulfide.

### Field experiments.

Oxygen (O_2_) depth profiles were measured with microsensors (detailed description of microsensor techniques below) at two different positions *in situ* and in two cores (consisting of >10 cm of sediment, mat, salt crust, and hypersaline water) in a nearby field laboratory. In the field and field laboratory, hardened salt crust was carefully removed in order not to break the sensor. Loose salt crystals were then added on top of the mat. Separate O_2_ measurements in cores were performed with artificial light (∼600 μmol · m^−2^ · s^−1^, KL1500 halogen lamp; Schott, Mainz, Germany) and natural illumination.

### Salinity shift laboratory experiments.

For transport, the water was drained from the cores and a headspace of one quarter of the core was left above the salt crust. The cores were transported upright in a dark box with coolant packs. The experiments at the MPI Bremen were conducted 7 days after the sampling. At SQU, Muscat, cores were inundated with water from the sampling site right after the sampling. The development of oxygen profiles upon salt dilution in the samples sent to the laboratory in Bremen were very similar to the samples kept in Oman, both in extent and temporal dynamics (Fig. 3A; see also Fig. S3 in the supplemental material), indicating that the microbial community was well preserved during transport.

At the MPI, microbial processes were first measured immediately upon arrival in moist cores that contained salt-saturated porewater and no water above the salt crust (Fig. S2). To study the effect of a strong tide or flood, the mats were inundated with filtered seawater (salinity, 3.5%) and kept under 12-h light (600 μmol · m^−2^ · s^−1^) and 12-h dark cycling conditions with continuous measurement of O_2_ profiles (see Fig. S4 in the supplemental material). The salt crust dissolved after 1.5 days, and after 3 days, the salinity in the upper mat (0 to 3 mm) dropped to 12% (Fig. S2). After all of the microsensor measurements on the inundated cores were finished, the water was left to evaporate for 3 days, resulting in 40% porewater salinity and a reformed salt crust (Fig. S2). The effects of these transitions on photosynthesis, respiration, and sulfate reduction, as well as photopigment distribution, were measured as described in detail below.

At SQU, two cores were inundated with water collected from the site (30% salinity) for 3 days, and O_2_ profiles under natural illumination were measured (see microsensor method description below). Subsequently, the water was carefully decanted and exchanged for 5% salinity (diluted water from the sampling site), and after 2 days, O_2_ profiles were measured again. The water was replaced with 30% salinity again, and O_2_ profiles were measured immediately and up to 90 min later.

### Microsensor measurements.

Microprofiles of O_2_, pH, and sulfide (H_2_S) were measured through the salt crust and into the mats in two cores to detect and quantify metabolic activities. Microsensors for O_2_, H_2_S, and pH were made, calibrated, and used as described previously (8, 68–70). At the MPI laboratory, a thin hole was made in the salt crust with a 0.4-mm needle mounted on the microsensor manipulator, through which the sensors were inserted. The sensors were mounted on a micromanipulator so that they penetrated at an angle of 40° to avoid shading of the studied area by the equipment. The mat surface was set as the 0-mm depth.

Local diffusive fluxes of O_2_ and H_2_S were calculated from three steady state microprofiles, and the change in fluxes with depth was used to calculate local conversion rates as described previously (71). The assumed diffusivities were 1.5 × 10^−9^ m^2^ s^−1^ for O_2_ and 1 × 10^−9^ m^2^ s^−1^ for total sulfur (S_tot_) (D_Stot_ = 0.64 × DO_2_) (7) and were corrected for a porosity of 0.3. Volumetric respiration and photosynthesis rates in Table 1 represent average values across mat depth where O_2_ concentrations were above zero.

### Geochemical analysis.

Sulfate reduction rates were determined by radiotracer incubation of sediments from the same cores as used for laboratory microsensor measurements at the MPI before (in triplicate) and 6 days after the inundation (in duplicate) (Fig. S1) in 5 ml cutoff syringes that were closed with a butyl rubber stopper. A volume of 25 μl of [^35^S]SO_4_^2−^ tracer solution (200 kBq per syringe core) was inserted in the syringe cores past the butyl rubber stopper. Sediments were incubated for 6.5 h in the dark at 26°C under an N_2_ atmosphere. To stop the reaction, sediment samples were transferred to 2 ml 20% (wt/vol) ZnAc and stored at −20°C. The top 3.2 mm (volume of 0.4 ml) was sampled, and below this depth, subsampling was conducted in 8-mm (volume of 1 ml) intervals. Further treatment of the samples and quantification of the rates were done as described previously (72). Blanks were directly transferred to 2 ml 20% (wt/vol) ZnAc after addition of 200 kBq of the tracer solution and were used to calculate the minimum detection limit (MDL) of the measurements.

Porewater sulfate and Cl^−^ were determined on parallel cores. Sampling was conducted as described above for sulfate reduction measurements. Subsamples were transferred to 15-ml centrifuge tubes and stored at −20°C until further processing. When overlaying water evaporated and a new salt crust formed (porewater salinity returned to 40%), sampling was repeated. All samples were transferred to Ultrafree-MC GV filter units with a 0.22-μm Durapore polyvinylidene difluoride (PVDF) membrane and centrifuged for 15 min at 14,000 rpm. Subsequently, retrieved porewater was transferred to Eppendorf tubes prefilled with 5% (wt/vol) ZnAc and stored at 4°C until further processing. Both sulfate and Cl^−^ concentrations were determined using ion chromatography (Metrohm 930 Compact IC Flex). Cl^−^ concentrations (μM) were converted to salinities (% wt/vol).

### Optical profiling.

Optical profiling to determine the layering of photopigments in the mats was performed at the MPI Bremen on two cores sampled in 2019 (Fig. S2). Mat cores were illuminated by artificial light with 600 μmol · m^−2^ · s^−1^ intensity (KL1500 halogen lamp; Schott, Mainz, Germany). Scalar irradiance optical microsensors (Zenzor, Denmark) were used to profile the light field within the salt crust, mat, and sediment layers (73). The salt layer was first penetrated with the optical fiber retracted into the needle to create a small hole to avoid damaging the spherical tip of the sensor probe. With the sensor extended out of the needle, the irradiance was measured every 400 μm within the salt, mat, and sediment layers using a spectrometer (USB2000+; Ocean Optics USA) attached to the other end of the optical fiber. Each spectrum was smoothened with a linear Savitzky-Golay filter (15-nm window, two passes) and compiled into a depth profile, with the zero-value set at the mat-salt interface. The irradiance spectra were normalized so that values represented the fraction of light at the salt-mat interface. Light intensity profiles were collected into the following three channels: UV (200 to 400 nm), photosynthetically active radiation (PAR) (400 to 700 nm), and near infrared (NIR) (750 to 950 nm). Spectral attenuation was calculated as the negative gradient between the consecutive log-transformed spectra of each profile. Absorbance peaks corresponding to photopigments chlorophyll *a* (Chl-*a*) (674 nm), phycocyanin (624 nm), bacteriochlorophyll *a* (BChl-*a*) (845 nm and 902 nm), and bacteriochlorophyll *c* (BChl-*c*) (745 nm) were identified in the attenuation spectra. The abundances of photopigments at a depth level were estimated as the area (if convex) under the attenuation curve within a 10-nm window around the peak absorption wavelengths.

### Hyperspectral imaging.

Hyperspectral imaging was done on the cores used for experiments at the MPI in 2019 (Fig. S2) in order to assess the lateral variability and depth distribution of photopigments within the mat. After the flooding followed by restoration of the salt crust, the core was subsampled to a depth of 5 cm using a syringe of 1 cm diameter with its tip cut off. The sample was sliced longitudinally to expose a cut section of the mat and scanned with a hyperspectral imager, as described previously (74), with a spatial resolution of ∼60 μm/pixel. The hyperspectral image, normalized to reflectance, was used to calculate the distribution of photopigment abundances by quantifying the absorption signals of photopigments in second-derivative spectral analysis (45, 75).

### Pigment extraction and measurement.

Mats sampled in the field in 2018 (Fig. S1) were sectioned in layers according to color as described above for the DNA and microscopy analyses. Three samples of each layer were freeze-dried and extracted twice with 1 ml ethanol/acetone/water (45:45:10) solution in each step in a 4°C cooled ultrasonic bath for 90 min each run. The supernatant was collected by centrifugation for 4 min at 16,000 × *g* (Eppendorf 5415C centrifuge) and stored at −20°C until analysis. Pigment extracts were then injected into a Waters ultraperformance liquid chromatography (UPLC) H-class system (Waters, Milford, MA, USA) equipped with an Acquity UPLC BEH C_18_ column (1.7 μm, 2.1 × 150 mm) (Waters Corporation, Milford, USA). Peak separation was based on an existing protocol (76) with gradients modified for UPLC instruments. The gradient started with 96% solvent A (75% methanol/25% water buffered with 0.5 M ammonium acetate) and 4% solvent B (90% acetonitrile/10% water) and increased linearly to 100% B at 0.75 min. At 2.63 min, 10% solvent C (100% ethyl acetate) was added and slightly increased over 40% (at 6.38 min) to 70% at 10.5 min and stayed constant to 12.59 min. Subsequently, the gradient was made 100% B at 12.97 min, and at 13.34 min, the column was resaturated with the initial mixture of A and B. The column temperature was 30°C, and the flow rate was constant at 0.2 ml/min. Data were analyzed with the software package Empower 3 (Waters, Milford, MA, USA). For total concentration, standards with defined concentrations were measured and response factors for all analyzed compounds were calculated.

### Microscopy.

For microscopic studies, 80 to 200 mg of PFA-fixed materials from different mat layers sampled in the field in 2018 (Fig. S2) were subjected to seven rounds of sonication with a UW 2070 probe (Bandelin Electronic, Berlin, Germany) (30 s at 20% power); after each round, 1 ml of supernatant was collected and replaced by 1 ml of ethanol/PBS (1:1) mixture. Collected supernatant was filtered on 0.2-μm pore-size polycarbonate filters (Sartorius, Göttingen, Germany), 30 μl per filter. The DNA of cells on filters was stained with 4′,6-diamidine-2′-phenylindole dihydrochloride (DAPI) and inspected under a fluorescence microscope. The number of cells per gram of mat (wet weight) was calculated based on DAPI signal counts. In filaments, individual cells delineated by visible septa were counted (see Fig. S5 in the supplemental material), not filaments as a whole. *Cyanobacteria* were identified by strong red autofluorescence of chlorophyll in the 660-nm channel in addition to strong orange autofluorescence of carotenoids in the 550-nm channel. Separate counts were made for cyanobacterial cells arranged in long filaments and large oval single cells. Further, cells with only orange autofluorescence in the 550-nm channel and filamentous morphology were counted as *Chloroflexi* (bacteriochlorophyll autofluorescence peaks at 790 nm and is not visible in the 660-nm channel). Cell area was calculated with ZEN Blue software based on manual identification of cell boundaries (Carl Zeiss, Jena, Germany).

### DNA extraction and sequencing.

DNA was extracted from each individual layer of three replicate mat patches. For each DNA extraction, ca. 400 mg of mat material from different layers sampled in 2018 was used. Before the extraction, the material was washed twice in 1× PBS (pH = 7.4) by resuspending, vortexing, spinning down at 10,000 × *g*, and removing the supernatant. The washed material was then extracted with a phenol-chloroform based protocol as described previously (86), which includes three rounds of extraction with bead-beating at maximum speed and removal of polymeric organic substances. The V4 region of the 16S rRNA gene was amplified by using the universal primers 515F-mod and 806R-mod with a broad coverage of *Bacteria* and *Archaea* (77) as described previously (78) and sequenced on Illumina MiSeq at the Vienna BioCenter Core Facility. DNA extraction and PCR amplification repeatedly failed for three samples (1× brown layer and 2× gray layer), likely due to failure to remove extracellular polymeric substances or other inhibitors. The obtained sequence reads were error-corrected using the SPAdes assembler (79), merged with BBmerge (80) and analyzed as described previously (81). Briefly, amplicon sequence variants were generated with DADA2 (82) and further clustered into operational taxonomic units (OTUs) by SWARM v2 (83). The centroid sequences of SWARM-generated OTUs were classified against the SILVA SSU132 non-edundant database (84) with the SINA aligner (85). All parameters were set as described in reference 81. Full data on relative sequence abundances are provided in File S1.

### Data availability.

All sequence reads have been uploaded to European Nucleotide Archive under the project accession number PRJEB37471.
