# Supplementary material for: Limitation of Microbial Processes at Saturation-Level Salinities in a Microbial Mat Covering a Coastal Salt Flat
Source: Appl Environ Microbiol. 2021 Aug 11;87(17):e00698-21. doi: 10.1128/AEM.00698-21 (PMC8357274; doi:10.1128/AEM.00698-21)
Supplement: Supplemental file 2 — Figures S1 to S7, Table S1. Download AEM.00698-21-s0001.pdf, PDF file, 1.7 MB [file aem.00698-21-s0001.pdf]

Limitation of microbial processes at saturation-level salinities in a microbial mat covering a coastal saltflat
-Supplementary material

Supplementary Figure 1

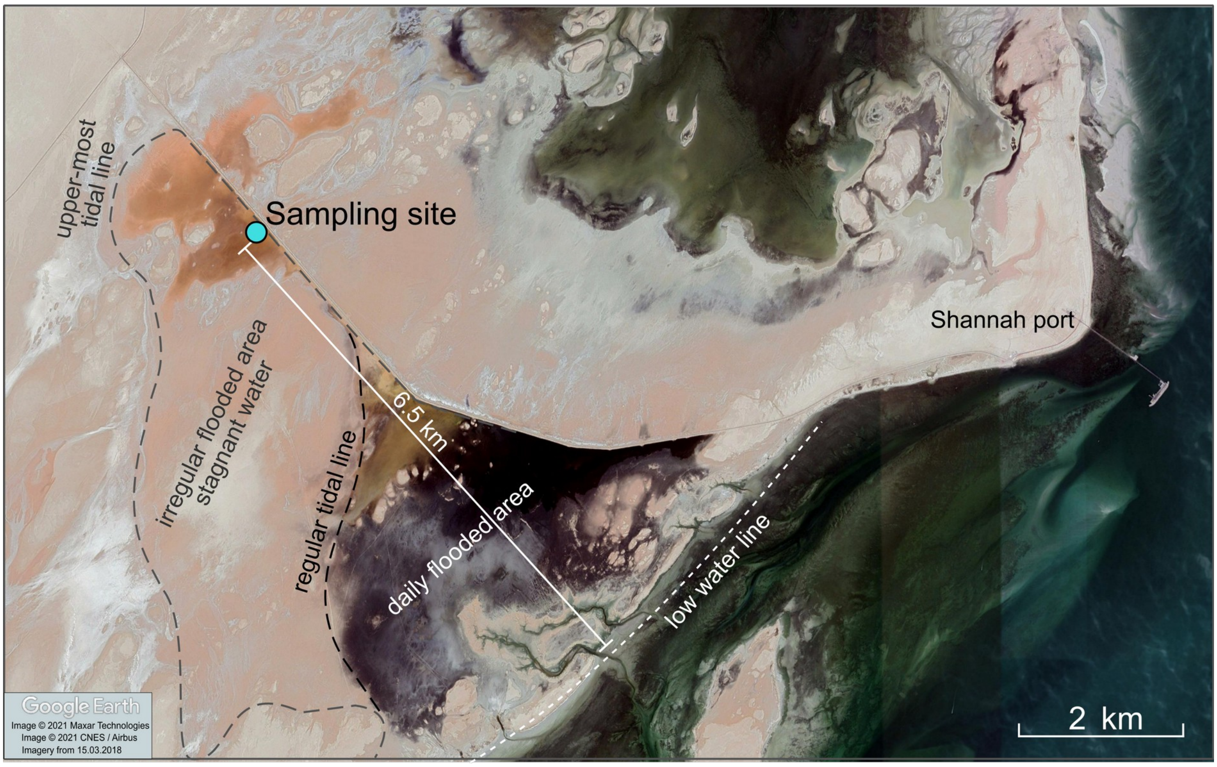

Figure S1: Satellite map of the tidal flat near Shannah. The coordinates of the sampling location are 20°45'39.6"N 58°38'52.3"E. Dashed lines indicate the approximate extents of daily flooded area and the more irregularly flooded areas, based on field observations. The map was generated with Google Earth using satellite imagery from 15.03.2018 (© 2021 Maxar Technologies, © 2021 CNES/Airbus).

Supplementary Figure 2

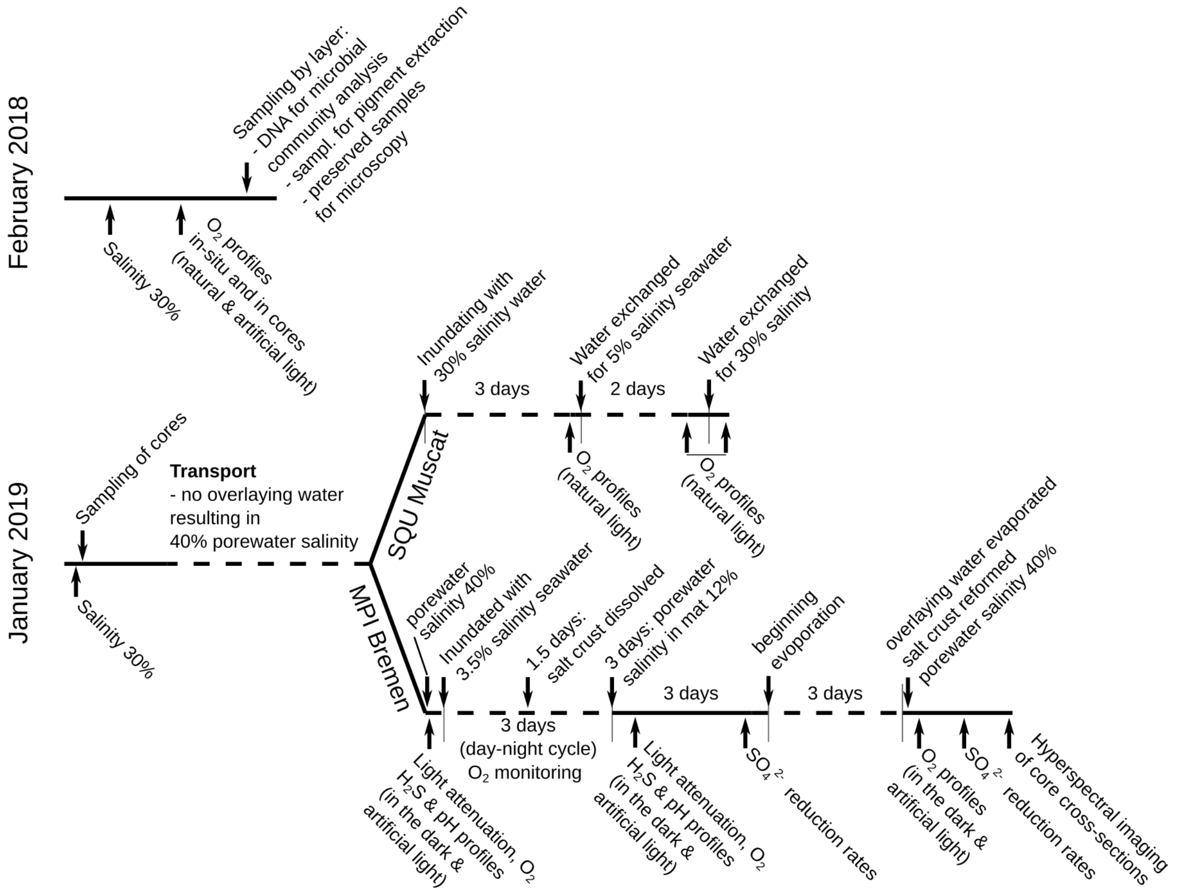

Figure S2: Overview scheme over the time course of experiments and measurements. The length of lines are not proportional to passed time. Therefore time spent at certain conditions is specifically indicated.

Supplementary Table 1: Ionic composition of the salt crust

All measurements were performed by AGES - Austrian Agency for Health and Food Safety Ltd.

Ion	Concentration (g/kg)
Cl	552
Na	355
SO_4	8.3
F	0.099
Ca	1.53
K	1.13
Mg	0.373
P	0.019
Zn	0.0049
Cu	0.0019
Fe	0.019
Mn	0.001

Chloride concentration was determined by the Volhard Method (AOAC 935.43).

Sulfate and fluoride were measured by HPLC-CD

All other elements were determined by Inductively-Coupled Plasma - Optical Emission Spectrometry

Supplementary Figure 3

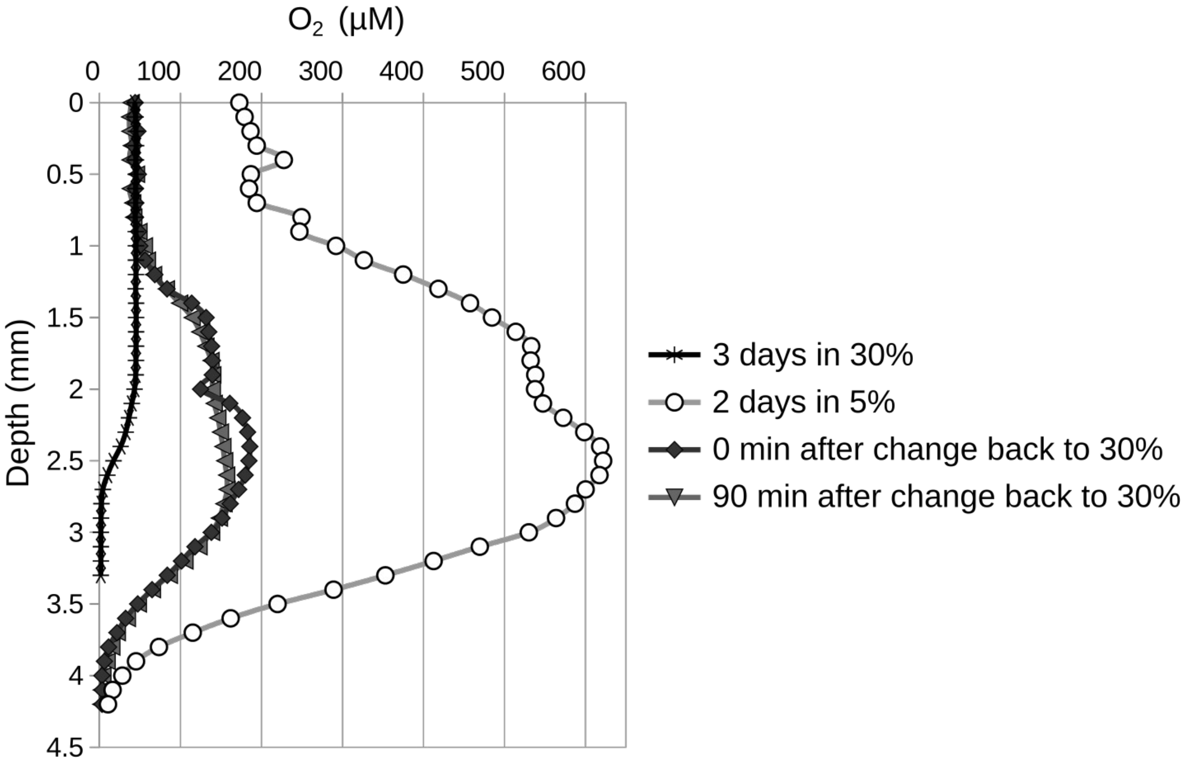

Figure S3: Profiles of oxygen concentrations in the mats sampled in January 2019. The profiles were measured in a core containing mat and underlying sediments brought to the laboratories of Sultan Qaboos University in Muscat, Oman. Upon arrival the core was flooded with 30% salinity water from the site for three days before measuring first profiles. Subsequently, the water was exchanged for 5% salinity water. After two days O_2 profiles were measured again. The water was exchanged back to 30% salinity and profiles were measured up to 90 min after the salinity increase. The figure shows representative profiles for clarity.

Supplementary Figure 4

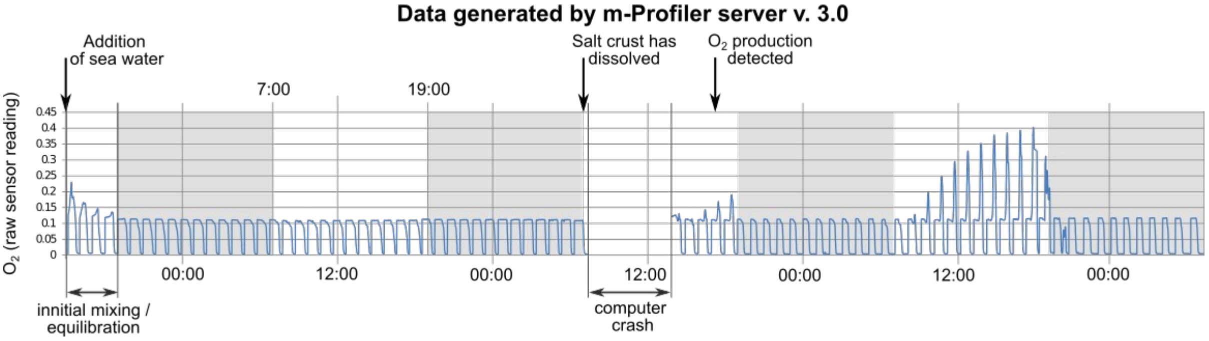

Figure S4: Log of the raw oxygen profiles after the inundation of a salt-saturated core in sea water. Oxygen profiles were measured continuously after pouring sea water on top of a salt crust-covered sediment and mat core. The core illumination (intensity 600 µmol·m^{-2}·s^{-1}) is indicated by white background. Dark stretches are indicated by grey background.

Supplementary Figure 5

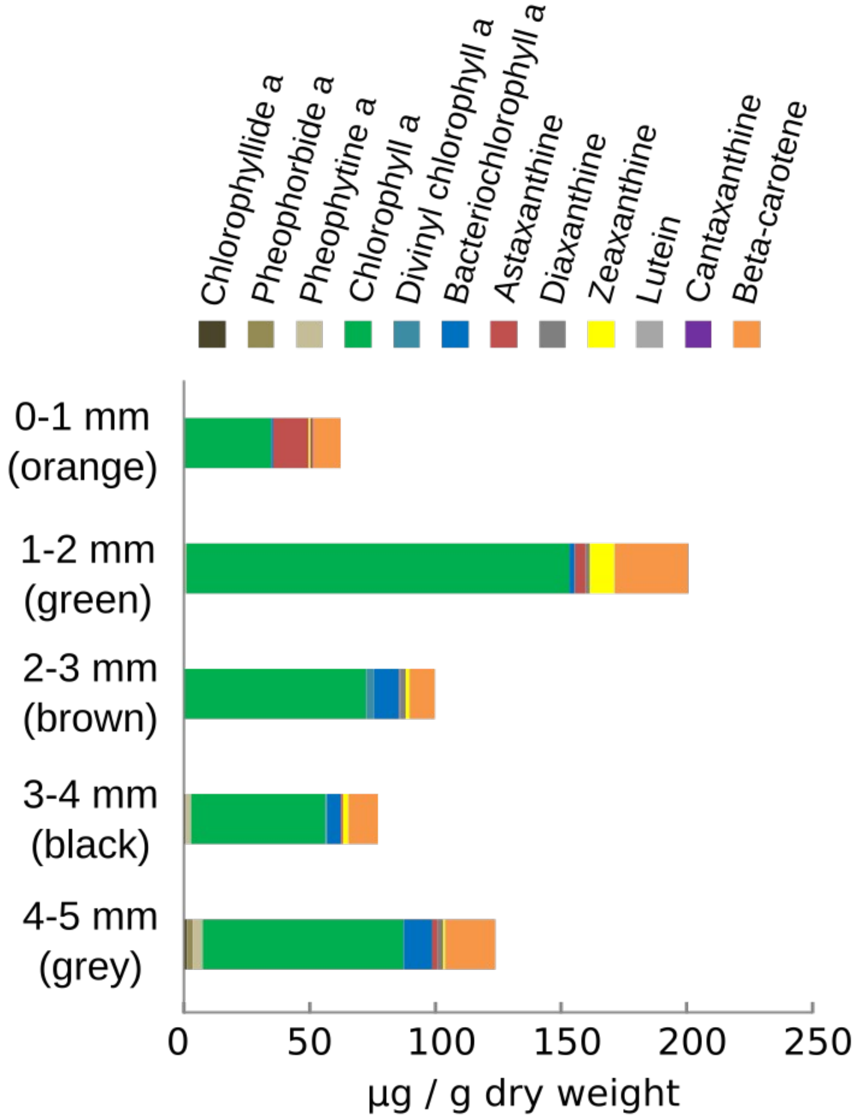

Figure S5: Average amount of pigments per gram dry weight in the five mat layers as determined by HPLC analysis. Chl-*a* shows highest abundance in the green layer. The grey layer had second highest Chl-*a* content. However, note also the slightly increased amounts of chlorophyll degradation products chlorophyllide a 126 and pheophorbide in the grey layer. Bchl-*a* becomes more abundant below 2 mm. In the upper peak zeaxanthin is found, which is absent in the lower peak. The values are averages from three replicate mat patches.

Supplementary Figure 6

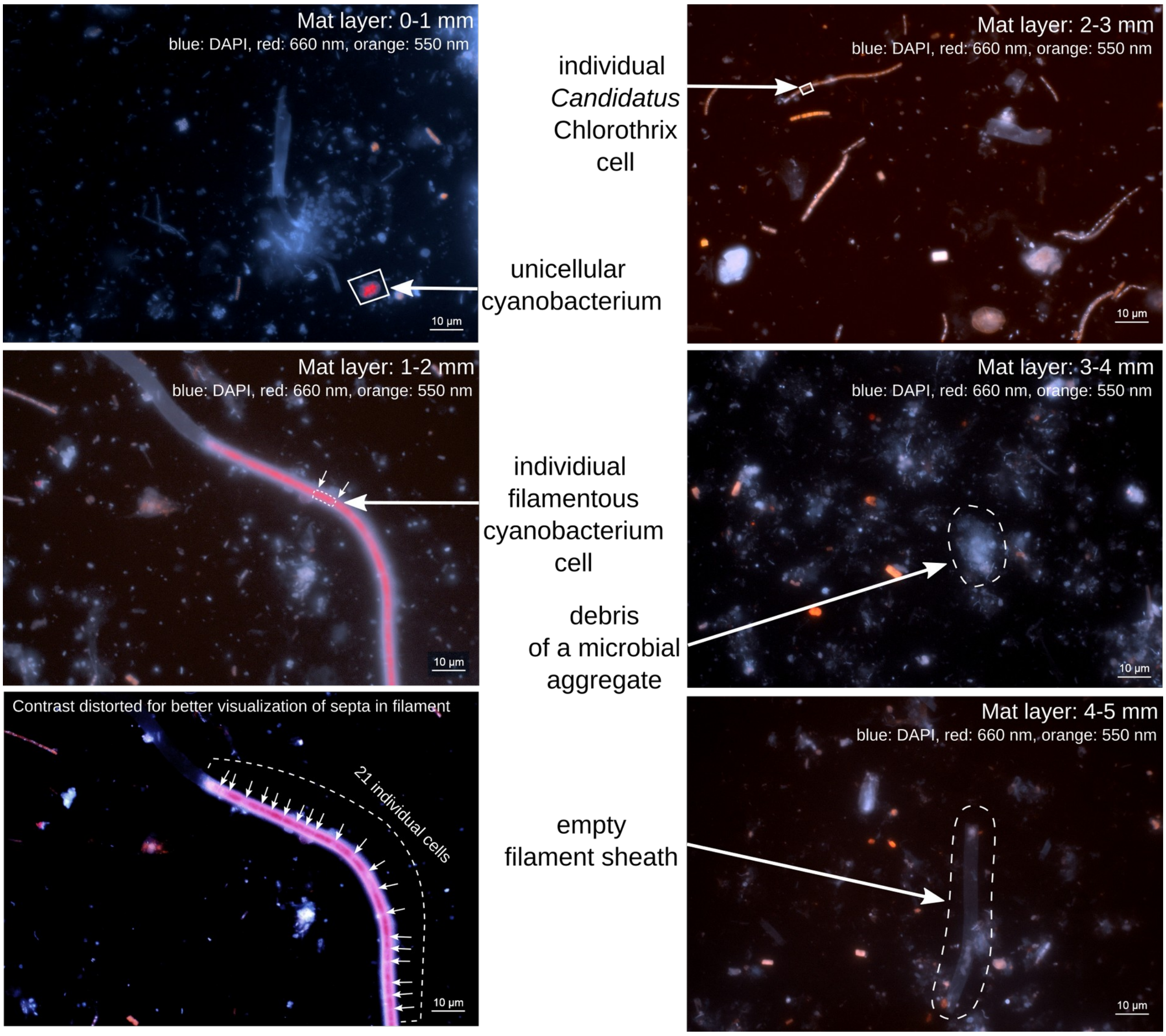

Figure S6: Example overlay images of fluorescence microscopy observation. The images illustrate objects counted as *Cyanobacteria* or *Chloroflexi* cells based on auto-fluorescence and morphology. For Cyanobacteria, only the red auto-fluorescence of Chl-a is shown (660 nm), as it was a distinctive feature used to identify them. Both *Chloroflexi* and *Cyanobacteria* had orange (550 nm) auto-fluorescence of carotenoids. In filaments, individual cells delineated by visible septa were counted, not filaments as a whole. Pictures were taken with 100x magnification on a Zeiss Axioplan Imager M1 with Zen Blue software (Carl Zeiss, Jena, Germany)

Supplementary Figure 7

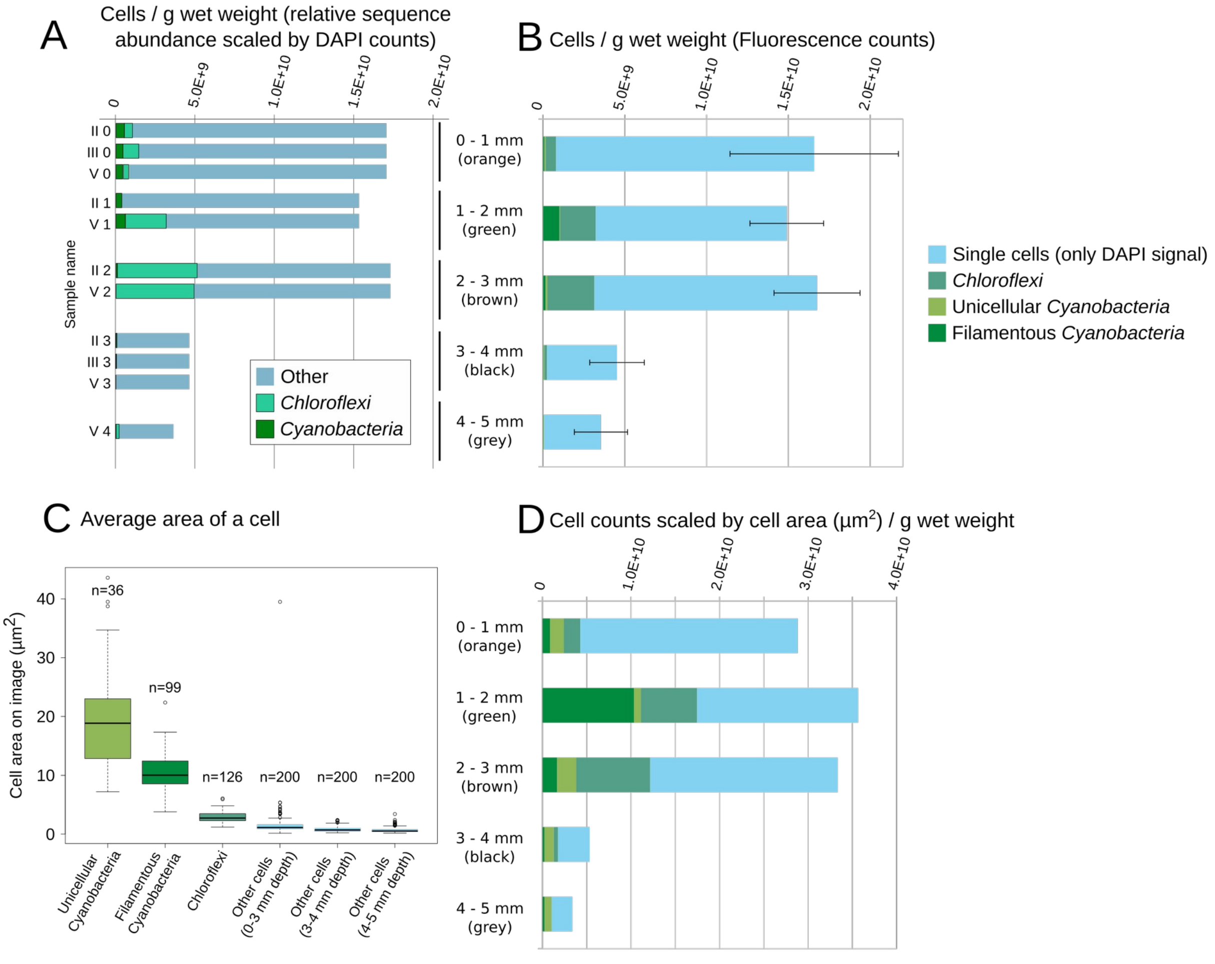

Figure S7: Cell numbers in different mat layers as determined by fluorescent microscopy. A) Abundances of *Chlorflexi* and *Cyanobacteria* based on 16S rRNA gene sequence data scaled by DAPI cell counts. B) Cell numbers by depth determined based on fluorescence counts on 10 microscopic images per depth. Total cell counts were determined by DAPI signal counts. *Cyanobacteria* and *Chloroflexi* counts were identified by auto-fluorescence of pigments (Chl-*a* and carotenoids) and morphology (examples in Fig. S6). **C)** Average areas of cells from the different counted groups. **D)** Proportion of biomass approximation, done by scaling the cell counts by the average cell area of the respective group. Note, that when scaled by volume the proportion of cyanobacterial biomass would be even significantly larger.
